# Overcoming Drug Resistance in a Clinical *C. albicans* Strain Using Photoactivated Curcumin as an Adjuvant

**DOI:** 10.3390/antibiotics12081230

**Published:** 2023-07-25

**Authors:** Carmen-Ecaterina Leferman, Laura Stoica, Mirela Tiglis, Bogdan Alexandru Stoica, Monica Hancianu, Alin Dumitru Ciubotaru, Delia Lidia Salaru, Aida Corina Badescu, Camelia-Margareta Bogdanici, Ioan-Adrian Ciureanu, Cristina-Mihaela Ghiciuc

**Affiliations:** 1 Department of Pharmacology, Medical Specialties II, “Grigore T. Popa” University of Medicine and Pharmacy, 700115 Iasi, Romania; carmen-ecaterina.leferman@umfiasi.ro (C.-E.L.);; 2 Department of Ophthalmology, “Grigore T. Popa” University of Medicine and Pharmacy, 700115 Iasi, Romania; 3 Department of Cell and Molecular Biology, “Grigore T. Popa” University of Medicine and Pharmacy, 700115 Iasi, Romania; 4 Department of Anesthesia and Intensive Care, Emergency Clinical Hospital of Bucharest, 014461 Bucharest, Romania; 5 Department of Biochemistry, “Grigore T. Popa” University of Medicine and Pharmacy, 700115 Iasi, Romania; 6 Department of Pharmacognosy, “Grigore T. Popa” University of Medicine and Pharmacy, 700115 Iasi, Romania; 7 Department of Neurology, “Grigore T. Popa” University of Medicine and Pharmacy, 700115 Iasi, Romania; 8 Institute of Cardiovascular Diseases, 700503 Iasi, Romania; 9 Department of Microbiology (Bacteriology, Virology) and Parasitology, “Grigore T. Popa” University of Medicine and Pharmacy, 700115 Iasi, Romania; 10 Department of Medical Informatics and Biostatistics, “Grigore T. Popa” University of Medicine and Pharmacy, 700115 Iasi, Romania

**Keywords:** PDT, fungi, *Candida albicans*, curcumin, antimicrobial photodynamic chemotherapy, efflux pumps, resistance

## Abstract

The limited antifungal drugs available and the rise of multidrug-resistant *Candida* species have made the efforts to improve antifungal therapies paramount. To this end, our research focused on the effect of a combined treatment between chemical and photodynamic therapy (PDT) towards a fluconazole-resistant clinical *Candida albicans* strain. The co-treatment of PDT and curcumin in various doses with fluconazole (FLC) had an inhibitory effect on the growth of the FLC-resistant hospital strain of *C. albicans* in both difusimetric and broth microdilution methods. The proliferation of the cells was inhibited in the presence of curcumin at 3.125 µM and FLC at 41 µM concentrations. The possible involvement of oxidative stress was analyzed by adding menadione and glutathione as a prooxidant and antioxidant, respectively. In addition, we examined the photoactivated curcumin effect on efflux pumps, a mechanism often linked to drug resistance. Nile Red accumulation assays were used to evaluate efflux pumps activity through fluorescence microscopy and spectrofluorometry. The results showed that photoactivated curcumin at 3.125 µM inhibited the transport of the fluorescent substrate that cells usually expel, indicating its potential in combating drug resistance. Overall, the findings suggest that curcumin, particularly when combined with PDT, can effectively inhibit the growth of FLC-resistant *C. albicans*, addressing the challenge of yeast resistance to azole antifungals through upregulating multidrug transporters.

## 1. Introduction

Annually, more than 150 million people worldwide are affected by fungal infections, making it a significant global health issue [1]. A large proportion of invasive fungal infections are linked to underlying health conditions that cause immunosuppression. Fungal infections can manifest a spectrum of severity, from superficial forms affecting the skin and nails to more serious, invasive, and disseminated infections that can be life-threatening [2]. Opportunistic fungal infections are predominantly caused by *Candida* spp., *Aspergillus* spp., and *Cryptococcus* spp., among others [2].

The antifungal agents regarded as clinically relevant are categorized based on their chemical structure and mechanism of action into five main groups: polyenes, azoles, allylamines, pyrimidines, and echinocandins [3]. The main action mechanisms of the antifungal agents target either the membrane (polyenes) directly or a component of the membrane (allylamines and azoles), while the β(1-3)-glucan synthase of the yeast cell wall is targeted by the echinocandins [3].

Researchers and clinicians have cataloged the therapeutic impacts of these drugs. Nonetheless, concerns regarding treatment-related toxicity and drug tolerability have arisen with their long-term use. It is crucial for clinicians to comprehend and evaluate adverse reactions to achieve therapeutic success. Echinocandins are the best-tolerated antifungal agents [4], while amphotericin B is the least tolerated, presenting numerous adverse effects that the clinician must consider, such as potassium level reduction, severe renal dysfunctions, and cardiac diseases, among others [5]. Triazoles, on the other hand, are mainly associated with gastrointestinal disorders, elevated liver enzymes, nervous system disorders, especially visual disturbances, and cardiac diseases [6].

The prophylactic use of antifungals has Increased as less toxic drug formulations have been developed to treat a variety of conditions in different patients. However, this reliance on antifungals as prophylactics and as a long-term therapy in high-risk patients has contributed to the emergence of multidrug-resistant fungi, such as a virulent strain *Candida auris* [7]. As drug resistance emerges, therapeutic options become limited. Multidrug resistance (MDR) can render treatment alternatives ineffective, adversely affecting patient outcomes.

Any class of antifungal drugs can encounter drug resistance that may manifest as an acquired resistance in strains known to be susceptible and others that are characteristically less sensitive. Resistance mechanisms related to drug efficacy may include several factors. These include the potential for modified interactions between drugs and their intended targets, diminished levels of drugs within cells due to the action of drug efflux transporters, and the presence of permeability barriers that are associated with biofilms [8,9,10].

Azole resistance is typically caused by an alteration or overexpression of the drug target gene in *Candida*-resistant isolates [11]. *C. albicans,* identified as the main fungal opportunistic pathogen affecting humans, is commonly found on the skin and mucosal membranes of healthy individuals [7].

However, it can cause superficial infections, which can develop into invasive candidiasis when the immune system of the host is compromised [2]. Despite aggressive antifungal treatments, *Candida* infections maintain a high mortality rate, mainly due to late diagnosis, the immunological state of the patient, and/or drug resistance [7].

While fluconazole (FLC) has been widely used in the treatment of *C. albicans* due to its broad efficacy profile and fungistatic activity, resistance can occur via several mechanisms. Among the most important ones to consider are modifications in the ergosterol biosynthesis pathway induced by mutagenesis or an overexpression of the ERG11 gene and active efflux of the administered compound [11,12,13].

The identification of potential drug targets to enhance the effect of FLC or even render it fungicidal has emerged as a promising approach. Limitations or failures of the conventional antifungal therapies are being answered by innovative synergistic combinations. The co-administration of chemical compounds (including natural ones) [14] and conventional chemotherapeutics is an interesting strategy for overcoming MDR in fungi [15].

Curcumin, a polyphenolic derivative found in *Curcuma longa*, is a fundamental component of turmeric and is used in Asian dietary and medicine traditions [16]. Human clinical studies have indicated its low toxicity with limited side effects, even at high dosage levels (up to 12 g/day) [17]. Biological functions of curcumin include anti-neoplastic, antioxidant, anti-inflammatory, antimicrobial, and hypoglycemic actions in human subjects [17].

Furthermore, the vast antifungal capacity of curcumin, specifically against *C. albicans*, has been documented [18]. It has demonstrated the ability to impede the formation and survival of fungal biofilms, a key element linked to resistance and repeated infections [19]. There is an increasing body of evidence indicating that these effects may be potentiated when applied synergistically with light [20,21].

Photodynamic therapy (PDT) is a minimally invasive technique that necessitates the activation of a photosensitive compound via visible light exposure [22]. The subsequent activation initiates a sequence of chemical reactions, culminating in the production of reactive oxygen species and other reactive molecules that trigger damage at the targeted biological location [23]. Initially conceived to address malignant conditions, the scope of PDT has expanded to include a wide range of disorders [24], including those of an infectious nature [25]. Antifungal PDT is a growing research interest, with a large part of the studies concentrated on in vitro experimentation [26]. This therapeutic strategy could present a potential alternative for handling fungal infections. The utilization of antifungal PDT in combating *C. albicans* and other *Candida* species has shown encouraging outcomes [20,21]. Since *Candida* species have already shown that they are sensitive to curcumin, the main purpose of this study was to see if the antifungal effects towards *C. albicans* could be improved by light exposure. The synergistic effects of curcumin with FLC against highly FLC-resistant clinical strains of *C. albicans* have already been established [14]. The most recognized mechanism of action correlates to the relationship between the efflux pumps and their involvement in the resistance to FLC.

Cdr1p and Cdr2p are ATP-binding cassette (ABC) transporters, powered by ATP hydrolysis, allowing them to transport a broad range of substrates, including many antifungal agents, across the cell membrane, often against a concentration gradient. Both Cdr1p and Cdr2p are heavily regulated at the transcriptional level, with their expression influenced by environmental factors and stress conditions, playing a critical role in the adaptation and survival of *C. albicans* in hostile environments. Since it has been reported as a substrate for the Cdr1p and Cdr2p heterologous efflux pumps in *S. cerevisiae* [27], our tested hypothesis was that curcumin could make it more sensitive to FLC treatment and that the resistance could be overcome through the characteristic photosensitivity of curcumin activated by PDT.

The microscopical and biochemical analyses of an FLC-resistant hospital strain of *C. albicans* showed the role of the efflux pumps in its resistance and investigated the photodynamic effect of curcumin against *C. albicans*.

## 2. Results

### 2.1. Determination of the Combined Effect of Fluconazole and Photo-Irradiated Curcumin Using the Difusimetric Method

**Figure 1:**
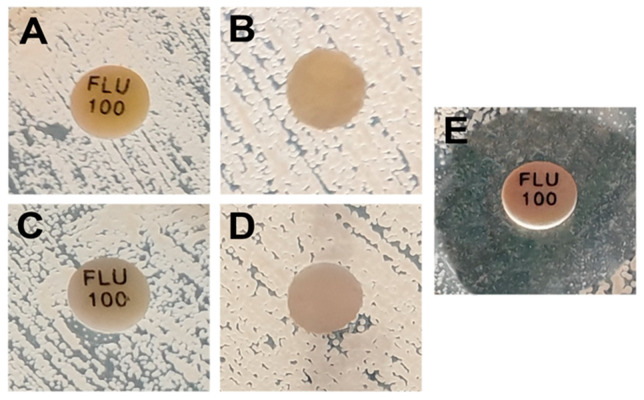
Difusimetric method showing the influence of simple and photo-irradiated curcumin on a fluconazole-resistant hospital *C. albicans* strain: (**A**)—fluconazole and curcumin, no light exposure; (**B**)—curcumin, no light exposure; (**C**)—fluconazole, light exposure; (**D**)—curcumin, light exposure; (**E**)—fluconazole and curcumin, light exposure. Concentrations: fluconazole 100 µg and curcumin 6 µM.

As shown in Figure 1, in the presence of 100 μg FLC, the cells showed resistance. Additionally, there is no effect of either the separated or combined treatment of curcumin and FLC on the growth of the resistant strain of *C. albicans* without light exposure. Only the co-treatment of cells with both FLC and curcumin (10 µL/disc, 6 µM concentration) exposed to PDT led to a significant inhibition of cell growth (Figure 1E). The inhibition diameter measured 21 ± 1 mm. Therefore, in the presence of light-exposed curcumin, FLC showed effective results in the inhibition of the resistant *C. albicans* strain.

### 2.2. Determination of the Combined Effect of Fluconazole and Photo-Irradiated Curcumin Using the Microdilution Method

**Scheme 1:**
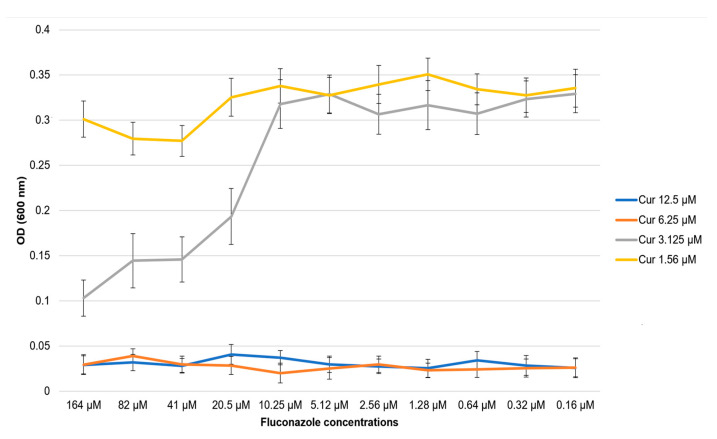
Influence of photo-irradiated curcumin at different concentrations on fluconazole-resistant *C. albicans* cell growth curves. The data represent the means of three independent experiments ± SE.

The effect of the combined treatment of curcumin and FLC was also determined by the serial twofold microdilution method in 96-well microtiter plates, when the plates were exposed to the light-emitting diode (LED) light source. Scheme 1 presents the PDT effect on the growth curves of the resistant *C. albicans* strain in the presence of different curcumin concentrations (12.5, 6.25, 3.125, and 1.56 μM) to which serial dilutions with FLC (from 164 to 0.16 µM) were added. In the presence of 3.125 μM curcumin, the cell growth was reduced by about 80% at 41 µM FLC. The phototoxicity of curcumin at 12.5 and 6.25 μM was high enough to inhibit the cell growth in all wells. At lower concentrations (1.56 μM or less), there was no inhibitory effect.

### 2.3. Influence of the Antioxidant/Prooxidant Addition on the Effect of the Co-Treatment of Fluconazole with Photo-Irradiate Curcumin

**Scheme 2:**
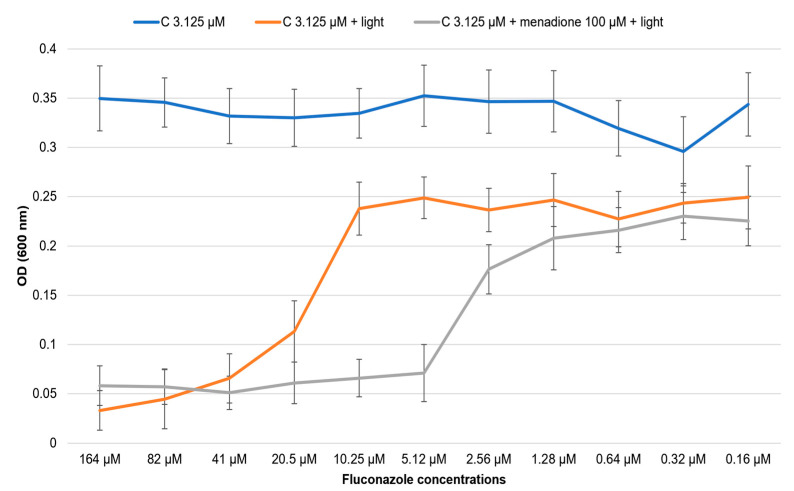
Influence of menadione (100 μM) on the photo-irradiated curcumin efficacy against fluconazole-resistant *C. albicans* cell growth curves (Curcumin (C) 3.125 μM was used in all experiments). The data represent the means of three independent experiments ± SE.

The addition of menadione at a 100 μM concentration, as a prooxidant compound, showed an enhancement in the cells’ growth inhibition effect of the co-treatment of fluconazole with 3.125 μM curcumin exposed to light (Scheme 2). The ability to inhibit 80% of yeast growth was lowered from a value of 41 to 5.12 μM of fluconazole.

The opposite effect was expected by the addition of glutathione as an antioxidant agent at 5 and 10 mM concentrations, as suggested by the literature data. The results showed an inhibitory yeast growth effect in all wells (data not shown). One possible explanation could be the interference with vitamin B12 from the medium, which is able to reverse the antioxidant effect of glutathione [28,29] and enhance the photochemical properties of curcumin.

### 2.4. Fluorescence Microscopy Assay for Monitoring the Influence of Photo-Irradiated Curcumin on Nile Red Accumulation

**Figure 2:**
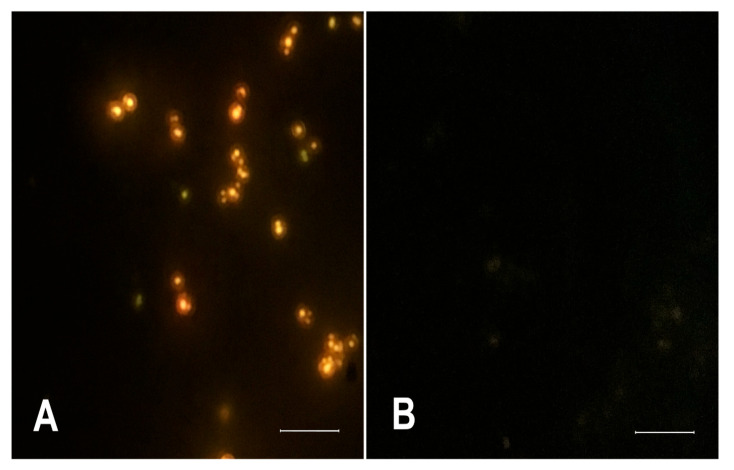
Nile Red fluorescence microscopy on fluconazole-resistant *C. albicans* strain in the presence of curcumin (3.125 μM): (**A**)—with light exposure; (**B**)—no light exposure. Bars represent 5 µm.

Fluorescent microscopy utilizing Nile Red revealed that it is removed from yeast cells by specialized efflux pumps [14]. Using Nile Red, which was reported as a substrate for Cdr1p, Cdr2p, and CaMDR1p [30], it was determined if the fluconazole-resistant *C. albicans* strain was capable of removing this compound [14,31]. *Candida* ATCC strains that have been treated with Nile Red were able to continue retaining this dye, whereas fluconazole-resistant cells exhibited only a slight fluorescence. Since a fluconazole-resistant *C. albicans* strain was able to release Nile Red, the effect of simple curcumin on the intracellular accumulation of this dye was examined and compared to the combined effects of curcumin and PDT. Hence, 3.125 μM of curcumin (as a previously effective tested concentration) was used in conjunction with the Nile Red staining technique. As illustrated in Figure 2A, cells treated with curcumin and PDT displayed substantially more visible fluorescence than cells not exposed to light in Figure 2B. Therefore, when exposed to PDT, curcumin was able to induce the intracellular accumulation of Nile Red.

### 2.5. Detection of Efflux Pumps Activity by Using Nile Red Efflux Assay

**Scheme 3:**
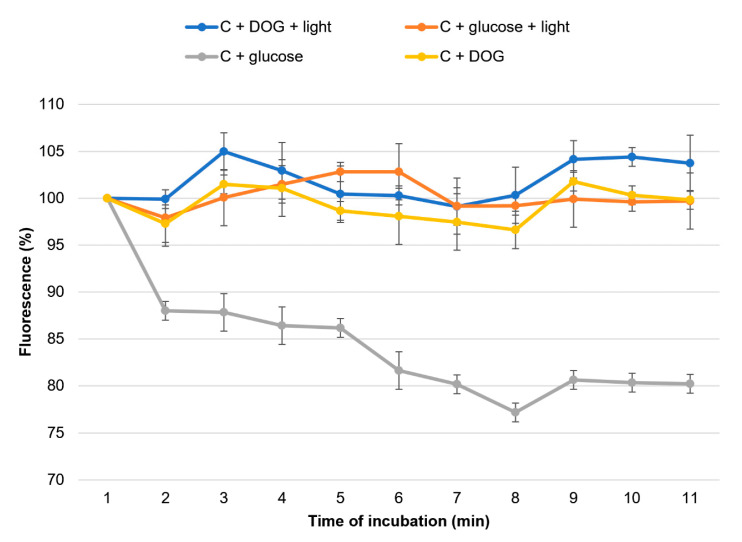
Influence of photo-irradiated curcumin on Nile Red efflux assay for fluconazole-resistant *C. albicans* cell growth curves (C—curcumin 3.125 μM; DOG—2-Deoxy-D-Glucose 50 μL, 80 mM). The data represent the means of three independent experiments ± SE.

The effect of small molecule inhibitors on glucose-dependent efflux has been studied previously using Nile Red and either CaMdr1p or CaCdr1p expressed in *S. cerevisiae* [29]. In the present study, Nile Red efflux was started by the addition of glucose (50 μL, 80 mM) at time 0 (with the exception of the blue and yellow lines, which represent cells to which 2-Deoxy-D-Glucose (DOG) was added instead of glucose) and in the end we measured cell-associated Nile Red fluorescence (Scheme 3).

After cells were energized, maximal Nile Red expulsion was reached in 7–8 min and this state of equilibrium lasted for at least a further 3 min (Scheme 3). As expected, the DOG-treated cells did not show a significant Nile Red efflux. In addition, the complete inhibition of energy-dependent pumping of Nile Red (fluorescence >95% of the initial value) was achieved in the presence of curcumin at 3.125 μM exposed to light at a total irradiance of 55 mW/cm^2^. These results suggest that the co-treatment of curcumin and PDT determines the inactivation of the efflux pumps as opposed to simple curcumin used at the same concentration.

## 3. Discussion

Chemotherapy has an important challenge, as MDR can manifest in a wide variety of clinical settings, from bacterial infections to cancer [32]. Due to the scarcity of antifungal agents that are currently available, MDR is a critical problem. Therefore, novel treatments to overcome this form of resistance, such as antifungal PDT coupled with the use of new drugs that target the efflux pumps, might be an option that is important to consider [33].

The photosensitization of *Candida* yeasts, which results in the induction of cellular damage, has been the focus of several different studies that used a variety of sensitizer chemicals [34,35]. A growing number of reports indicate that *Candida* can be resistant to antifungal drugs such as azoles [32,36]. The impact of PDT has previously been documented in the prevention of germ tube formation [34,37], decrease in adherence to epithelial buccal cells [38] and biofilm formation [37,39]. The fungicidal effect of PDT may depend on the strain, and although PDT was effective against *Candida* species, FLC-resistant strains exhibited a lower susceptibility to PDT [39].

To investigate the antifungal effect of PDT mediated by curcumin, this natural compound was associated with irradiation using an LED light source with a total irradiance of 55 mW/cm^2^ (exposure time of 30 min). The co-treatment of curcumin and FLC with PDT had an inhibitory effect on the growth of the FLC-resistant hospital strain of *C. albicans* in both difusimetric and broth microdilution methods. In contrast, there was no effect of the separated or combined treatment of curcumin and FLC on the growth of the resistant strain of *C. albicans* at the doses used in our study.

On the other hand, curcumin by itself has been proven to have antifungal activity [40]. It was demonstrated through broth microdilution that the growth of *Candida* cells was inhibited at doses of 185 mg/L or lower, but higher values of 296–370 mg/L were required to inhibit the growth on solid medium [41]. In another study, *C. albicans* was subjected to curcumin concentrations ranging from 1.56 to 12.5 g/L [42]. Therefore, the concentration at which curcumin would be effective seems to be substantially higher when compared with the doses that were employed in the current experiment, when used in combination with PDT. This is most likely due to the low concentration that was required for photoactivation.

In the current study, our focus was the PDT effect on the growth curves of the *C. albicans* strain, when using various doses of curcumin (12.5, 6.25, 3.125, and 1.56 µM) to which successive dilutions of FLC (from 164 to 0.16 µM) were added. The proliferation of the cells was inhibited by 80% in the presence of curcumin at 3.125 µM and FLC at 41 µM concentrations. The results showed that the phototoxicity of curcumin at concentrations higher than 6.25 µM combined with FLC inhibits yeast cell growth in every well. There was no inhibitory effect when the concentration was decreased to 1.56 µM. The combination of chemical therapy (curcumin and FLC) and PDT has proven to be effective for the cell growth inhibition of *Candida albicans*. These results are in line with a previous study conclusion, where 5 μM curcumin and 9 J/cm^2^ of blue light were used, eradicating *C. albicans* colonies [21]. Other comparable studies have reported the effectiveness of the PDT with 40 μM curcumin and blue light [20,43].

Another variable that has been shown to influence the uptake of the photosensitizer (PS) by the cells and the subsequent survival of the yeast is pre-irradiation time (PIT) [20]. In our experiment, the well plate was subjected immediately to LED light in order to prevent PDT effectiveness due to PIT.

The mode of action of PDT is determined by the visible light photons of a certain wavelength interacting with the intracellular molecules of the PS [22]. The reaction results in the production of reactive species, which in turn causes oxidative stress [22]. This causes a disruption in the equilibrium between prooxidants and antioxidants, shifting the balance in favor of the former and potentially causing damage. It seems that the excited states of curcumin and their subsequent chemical interactions with oxygen are responsible for mediating the phototoxicity of curcumin to fungal organisms [20,44].

In the present study, the role of oxidative stress in the inhibitory effect of photoactivated curcumin was tested through the addition of an antioxidant and a prooxidant compound, respectively. As expected, the addition of menadione at a 100 μM concentration, as a prooxidant compound, showed an enhancement in the cell growth inhibition effect of the co-treatment of FLC with 3.125 μM curcumin exposed to light. The necessary concentration of FLC needed to inhibit 80% of yeast growth was lowered to a value of 5.12 µM. The opposite effect was expected by the addition of glutathione as an antioxidant agent at 5 and 10 mM concentrations, as suggested by the literature data [45,46]. However, the results showed an inhibitory yeast growth effect in all wells at both concentrations. A possible explanation could be that vitamin B12, a component of RPMI 1640 medium, may disrupt the known antioxidant properties of glutathione and enhance the photochemical properties of curcumin [28,29].

Multiple FLC-resistant *C. albicans* highly express the *MDR1* gene, which encodes a membrane transport protein from the major facilitator superfamily (MFS) [47]. Members of this family of efflux pumps lead to a reduction in intracellular drug accumulation [48]. Moreover, when the MDR1 gene is removed from these overexpressing strains, their resistance to fluconazole decreases, indicating that the upregulation of MDR1 plays a crucial role in their drug resistance [47].

The effect of photo-irradiated curcumin on efflux pumps was investigated through fluorescence microscopy and biochemical tests. The possible link between the hospital strain resistance to FLC and the overexpression of the efflux pumps was evaluated by measuring the capacity of the strain to retain Nile Red dye. It has been previously established that *C. albicans* ATCC strains treated with Nile Red have been shown to be able to retain this dye, but FLC-resistant cells exhibit a less intense fluorescence [14,30]. The results of the present study showed that photosensitized curcumin at a concentration of 3.125 μM demonstrated the capacity to have an inhibitory action on the active transport of the fluorescent substance that is effluxed from cells. Another study indicated that the combination of 11 μM of simple curcumin with FLC at 4 mg/L was capable of inhibiting the growth of a clinical isolate of *C. albicans* and indicated that the efflux pumps could be a factor and contribute to the resistance of that strain to FLC [14].

The effect of small molecule inhibitors on a glucose-dependent efflux was previously studied with the help of Nile Red as a substrate for multidrug transporters overexpressed in *S. cerevisiae* [31]. In the current investigation, the evaluation of the activity of glucose-dependent efflux pumps showed the ability of photoactivated curcumin to inhibit pump-mediated Nile Red efflux. The release of Nile Red was activated by glucose addition (50 μL, 80 mM) at time 0. The maximum amount of Nile Red exported from the energized cells was reached after 7 to 8 min. As predicted, the cells that had been treated with DOG did not exhibit a substantial Nile Red outflow. In addition, the energy-dependent transport of Nile Red was completely blocked (fluorescence was more than 95% of the original value) in the presence of photoactivated curcumin at a concentration of 3.125 μM. As a result, there was an increase in effectiveness, deactivating the efflux pumps, at a similar dosage of curcumin when used with PDT.

According to the findings of this research, curcumin is not only a naturally occurring molecule that is capable of suppressing the development of *C. albicans*, but it is also capable, at low dosages, of acting in concert with FLC and PDT as a cell growth inhibitor of a strain of *C. albicans* resistant to FLC.

## 4. Materials and Methods

### 4.1. Strain and Media

The microorganism used in this study was an azole-resistant hospital strain of *C. albicans* (including fluconazole), kindly offered by Aida Badescu from the Department of Infectious Diseases, University of Medicine and Pharmacy of Iasi, Romania.

The media used in this work were RPMI 1640 (Gibco, Bethesda, MD, USA) supplemented with glucose (2% final concentration). The *C. albicans* strain was grown in RPMI-supplemented medium at 35 °C with constant shaking (200 rpm).

Maintenance and preservation of microorganisms: The clinical isolate was long-term stored in 20% glycerol at −20 °C and later subcultured on Sabouraud agar. The working strain was maintained and stored on Sabouraud dextrose agar (SDA) slants at 4 °C.

### 4.2. Reagents

Curcumin, fluconazole, reduced glutathione, menadione, Nile Blue, and dimethyl sulfoxide (DMSO) were obtained from Sigma Aldrich (St. Louis, MO, USA). Fluconazole disks (100 µg) were purchased from Liofilchem, Italy.

Nile Red was prepared from Nile Blue using the following steps: boiling a solution of 1% Nile Blue (2 h under reflux in 0.5% H_2_SO_4_), extraction of the product into xylene, separation, filtration and recrystallization [48].

Preparation of stock and working solutions: Stock solutions of various concentrations were dissolved in dimethyl sulfoxide (DMSO) for those not soluble in the used media, taking into account a maximum of 1% DMSO in the working solutions.

The curcumin solutions were prepared to a stock concentration of 5 mM using DMSO as a solvent and the final tested concentrations ranged from 60 to 1.56 µM by dilution with RPMI-supplemented medium.

The fluconazole stock solution contained 100 mg/mL compound in DMSO and the final concentrations ranged between 50 and 0.05 µg/mL (164 to 0.16 µM).

Menadione was dissolved in DMSO (10 mM) and used at 100 µM. A reduced form of glutathione (GSH) was prepared from a stock solution of 0.5 M and used at 10 and 5 mM.

### 4.3. Light Source for PDT

A light-emitting diode (LED)-based device, composed of two LED matrices (12/12), was used to excite the curcumin. This LED device provided a uniform emission of white light, including wavelengths from 440 nm to 460 nm. The total irradiance delivered was 55 mW/cm^2^. The total irradiation time was 30 min with constant ventilation.

### 4.4. Determination of the Combined Effect of Fluconazole and Photo-Irradiated Curcumin Using the Difusimetric Method

The *C. albicans* strain was cultured in 20 mL of RPMI broth overnight for a duration of 20 h. The culture was then diluted to achieve an optical density of 0.1 at a wavelength of 600 nm (OD_600_). The diluted *Candida* strain was spread using sterile Dynarex cotton-tipped applicators at perpendicular 90° angles. After allowing 10 min for drying, cotton disks measuring 6.6 mm in diameter and containing 100 μg of FLC were gently placed on the surface of the Sabouraud solid medium without disrupting the gel. A working solution of curcumin, with a volume of 10 µL and a concentration of 6 µM, was applied either on top of the fluconazole disks or on simple cotton disks measuring 6.6 mm in diameter. The cultures were incubated for 48 h at a temperature of 35 °C, and all treatments were performed in triplicate.

### 4.5. Determination of the Combined Effect of Fluconazole and Photo-Irradiated Curcumin Using the Microdilution Method

The effect of combining simple or photoactivated curcumin with fluconazole was assessed using a modified version of the Clinical Laboratory Standards Institute (CLSI) M27-A2 protocol [49]. In summary, yeast cells were cultured overnight in RPMI broth at 35 °C with constant agitation (250 rpm). From this culture, a concentration of 4 × 10^3^ cells/mL was added to the wells of a 96-well microtiter plate containing RPMI medium. The fluconazole concentration in the medium was twofold diluted, and various concentrations of curcumin were included. The minimum inhibitory concentrations (MICs) were determined after 24 h of incubation at 35 °C. MICs were defined as the drug concentration at which a decrease of 80% in turbidity was detected compared to the growth control without drugs. The experiments were conducted in triplicate, both with and without light irradiation.

### 4.6. Influence of the Antioxidant/Prooxidant Addition

The same conditions were kept as above, with a single curcumin concentration of 3.125 µM. Reduced glutathione (10 and 5 mM) or menadione (100 µM) were added to the final solutions prior to the plates irradiation.

### 4.7. Nile Red Accumulation Assay

To perform accumulation assays, the cells were cultivated overnight in RPMI medium at a temperature of 35 °C with continuous agitation. Following incubation, the cells were harvested through centrifugation at 4500× *g* for 5 min at 4 °C and were subsequently washed twice with a 10 mM phosphate buffer solution (PBS). A final cell density of 2 × 10^6^ cells/mL was achieved by suspending the cells in 1.0 mL of PBS supplemented with 2% glucose. To assess the ability of curcumin to induce Nile Red accumulation, the cells were incubated at a temperature of 30 °C for 30 min in the presence of curcumin at a concentration of 3.125 μM, with or without light irradiation. Nile Red dye at a concentration of 7 μM was then added to the cells [14]. Afterward, all cells were centrifuged at 4500× *g* for 5 min at 4 °C and washed twice with cold PBS. The final cell pellet was resuspended in 1 mL of PBS, and fluorescence microscopy analysis was performed using an Olympus BX51 fluorescence microscope from Tokyo, Japan.

### 4.8. Nile Red Efflux Assay

To evaluate the pump efflux activity of *C. albicans* cells, a previously established Nile Red pumping assay was employed [50]. After overnight growth to an optical density of approximately OD_600_ ~2–3, the cells were rinsed with sterile PBS, resuspended in PBS at an optical density of OD_600_ ~2, and kept on ice overnight to decrease the intracellular ATP levels. Subsequently, the starved cells were washed, resuspended at an optical density of OD_600_ ~1 in 5 mM 2-Deoxy-D-Glucose, and incubated with gentle agitation at a temperature of 30 °C for 30 min. Following this, Nile Red dye at a concentration of 7.5 μM was added to the treated cells and incubated for 30 min at 30 °C. The cells were then washed twice with PBS, resuspended at an optical density of OD_600_ = 10, and maintained on ice for a duration of 2 h. In 96-well microtiter plates, 50 μL of the cell suspensions were mixed with 50 μL of either 80 mM glucose or 80 mM DOG (as a control) in each well. In the presence of glucose, normal *C. albicans* cells would actively pump the dye out of the cells, resulting in a decline in fluorescence. The pumping assays were performed in triplicate in the presence of 3.125 μM curcumin and either with or without light irradiation. Fluorescence measurements were made using a Genios Tecan (Grödig, Austria) fluorimeter with an excitation filter at 485 nm and an emission filter at 528 nm. The initial measurement was captured 1 min after the addition of glucose (T0), and subsequent measurements were recorded for 11 min, with 15 s of shaking between each measurement.

## 5. Conclusions

In conclusion, curcumin demonstrated potential as a PDT agent, effectively inhibiting the growth of a fluconazole-resistant clinical strain of *C. albicans* with superior results than curcumin alone. Since it is well accepted that the upregulation of multidrug transporters is one of the primary mechanisms by which human pathogenic yeast acquires resistance to azole antifungals, our results are an attempt to address this problem and provide a possible solution in the form of an adjuvant therapy.

## Data Availability

Not applicable.
